# Inactivated Sendai virus strain Tianjin, a novel genotype of Sendai virus, inhibits growth of murine colon carcinoma through inducing immune responses and apoptosis

**DOI:** 10.1186/1479-5876-11-205

**Published:** 2013-09-05

**Authors:** Liying Shi, Jun Chen, Qiping Zhong, Mei Li, Peng Geng, Jianmin He, Zhe Han, Mingwei Sheng, Hua Tang

**Affiliations:** 1 Department of Microbiology, Basic Medical College, Tianjin Medical University, Tianjin 300070, China; 2 Department of Anesthesiology, Tianjin First Center Hospital, Tianjin 300192, China

**Keywords:** Sendai virus strain Tianjin, Murine colon carcinoma, Antitumor immunity, Dendritic cells, Apoptosis

## Abstract

**Background:**

Ultraviolet-inactivated, replication-defective Sendai virus particles (Z strain) have displayed antitumor effect through enhancing the immune responses or inducing apoptosis in a variety of carcinomas. Sendai virus strain Tianjin was isolated from the lungs of marmoset and proved to be a novel genotype of Sendai virus. In this study, we explored the antitumor effect and its mechanism of ultraviolet-inactivated, replication-defective Sendai virus strain Tianjin (UV-Tianjin) in mice bearing CT26 colon carcinoma.

**Methods:**

Three injections of UV-Tianjin were delivered into CT26 tumors growing on the back of BALB/c mice. Tumor size was measured in a blinded manner and survival rate of mice was calculated. In order to make clear antitumor mechanism of UV-Tianjin, the maturation and interleukin-6 (IL-6) release from murine myeloid dendritic cells (DCs) was examined by flow cytometry or ELISA assay after induced by UV-Tianjin and compared with those of live virus. Moreover, real-time RT-PCR and immunohistochemistry was performed to identify whether UV-Tianjin could induce infiltration of DCs, CD4^+^ and CD8^+^ T cells into tumors. The TUNEL assay was done to observe the apoptosis of CT26 tumor cells after UV-Tianjin injection.

**Results:**

In animal model, UV-Tianjin could obviously inhibit the growth of CT26 tumors and prolong the survival of the tumor-bearing mice compared with control group (*P* < 0.01). *In vitro* murine DCs stimulated by UV-Tianjin underwent dose-dependent maturation, similar to that elicited by live virus. And the secretion amount of IL-6 from DCs induced by UV-Tianjin was a little lower than that released in the presence of live virus. Real-time RT-PCR and immunohistochemistry revealed that UV-Tianjin induced a remarkable infiltration of DCs, CD4^+^ and CD8^+^ T cells into tumors. The TUNEL assay showed that the apoptosis index of tumor tissues injected with UV-Tianjin was significantly higher than that of control group (*P* < 0.01).

**Conclusions:**

Our results have demonstrated that UV-Tianjin alone could inhibit the growth of CT26 tumor in mice through enhancing host antitumor immunity and inducing apoptosis of tumor cells. Therefore, UV-Tianjin shows its prospect as a novel drug for carcinoma therapy.

## Background

Cancer has been one of the most serious diseases threatening to human health. Although the mortality of cancer patients has improved by operation, chemotherapy and radiotherapy, some advanced or metastatic tumors remain refractory to conventional treatment. Therefore, novel therapeutic approaches are urgently required to treat refractory tumors.

It is known for several decades that some viruses may be able to eradicate tumors. For example, adenovirus vector containing P53 gene has passed through clinical trials in many countries and proved to have therapeutic effects on various tumors [1,2]. Moreover, a variety of live viruses such as mumps virus, measles virus, Newcastle disease virus (NDV), reovirus and vesicular stomatitis virus (VSV) have been used for cancer treatment due to their ability of killing tumor cells selectively [3-7]. Sendai virus (SeV; also known as hemagglutinating virus of Japan, HVJ) is a member of the genus *Respirovirus* within the family *Paramyxoviridae*. It usually causes outbreaks of lethal pneumonia in mouse colonies, the natural host, but is thought to be nonpathogenic to humans. UV-inactivated Sendai virus (called HVJ-envelope, HVJ-E) is unable to replicate in cells because the genome is destroyed by UV irradiation, but conserves the complete structure of the live HVJ envelope containing F, HN, and M proteins [8]. Therefore, HVJ-E is safe and has been applied to an anticancer vaccine strategy [9,10]. Recent studies have demonstrated that HVJ-E (Z strain) has an extensive antitumor role and could eradicate or inhibit the growth of various tumors, including colon carcinoma, renal carcinoma, prostate cancer, malignant glioma, and melanoma through inducing antitumor immunity or tumor cell apoptosis [11-15]. However, the detailed mechanism of the antitumor activity of HVJ-E is not fully understood.

Sendai virus strain Tianjin (GenBank: EF679198.1) was isolated from the lungs of marmoset with respiratory infection in 1999. Phylogenetic analysis based on complete sequence has proved that Tianjin strain was a novel genotype of Sendai virus [16]. Our previous study has shown that non-replicating Tianjin strain could enhance the immune response through inducing the production of IFN-α, IL-6 and TNF-α *in vivo*[17]. This study was designed to demonstrate that UV-inactivated Tianjin strain alone may suppress colon carcinoma growing in mice through inducing not only immune response but also tumor cell apoptosis, which may be useful in developing viral therapeutics for cancer.

## Materials and methods

### Cell lines and mice

The CT26 murine colon carcinoma cell line was purchased from Cell Bank of Chinese Academy of Science (Shanghai, China). The cell line was maintained in RPMI 1640 supplemented with 10% FCS (Hyclone, Beijing, China), 100 U/ml penicillin and 100 U/ml streptomycin under a humidified atmosphere containing 5% CO_2_ at 37°C. Six-week-old, female BALB/c mice purchased from Beijing HFK Bioscience Co., Ltd. (Beijing, China), were maintained in a temperature-controlled and pathogen-free room. All animals were handled according to the approved protocols and the guidelines of the Animal Committee of Tianjin Medical University.

### Reagents and antibodies

Recombinant mouse IL-4 and GM-CSF were purchased from PeproTech Inc. (Suzhou, China). PE anti-mouse CD11c, FITC anti-mouse CD40, CD80, CD86 antibodies, PE Armenian Hamster IgG Isotype Ctrl, FITC Rat IgG2α, κ Isotype Ctrl, FITC Armenian Hamster IgG Isotype Ctrl were purchased from BioLegend (Beijing, China). Mouse IL-6 ELISA kit was obtained from RayBiotech (Guangzhou, China). Anti-CD11c antibody was purchased from Abcam Ltd (Hong Kong, China). Anti-CD4 antibody was purchased from R&D Systems China Co. Ltd. (Shanghai, China). Anti-CD8α antibody was from Santa Cruz Biotechnology Inc. (Beijing, China). TUNEL apoptosis assay kit was purchased from Roche Applied Science (Hong Kong, China).

### Preparation of live Tianjin strain and UV-inactivated Tianjin strain

Tianjin strain (GenBank: EF679198.1) was preserved in our laboratory and propagated in the chorioallantoic fluid of 10-day-old embryonated chicken eggs, after which it was purified by centrifugation and inactivated by UV irradiation (99 mJ/cm^2^), as previously described [8]. Inactivated virus is unable to replicate because the genome is destroyed by UV irradiation, but its capacity for viral fusion remains intact.

### Preparation and culture of dendritic cells

Dendritic cells (DCs) were generated from the bone marrow precursors of BALB/c mice as described in reference [18]. In brief, bone marrow of the tibia and femur was flushed with culture medium and treated with RBC lysis buffer for 5 min, washed twice with PBS, then cultured in complete RPMI 1640 medium supplemented with 20 ng/ml recombinant mouse GM-CSF and 20 ng/ml recombinant mouse IL-4. Half of the medium was replaced at day 2 and 4. At day 6, the suspended and loosely adherent cells were collected and CD11c molecule specific for DC was analyzed by flow cytometry. These cells were used in subsequent experiments as immature dendritic cells.

### Flow cytometric analysis

Immature murine DCs (1 × 10^6^ cells/ml) were placed in 6‒well plates and cultured with different doses of UV-inactivated Tianjin strain or live virus for 48 h. Then the DCs were incubated with anti‒mouse CD40, CD80, and CD86 antibodies, respectively. The stained cells were then analyzed with the BD FACSCalibur system and CellQuest software (BD Biosciences).

### Cytokine measurements

Immature murine DCs (1 × 10^6^ cells/ml) were cultured in 96‒well plates and then different doses of UV-inactivated Tianjin strain or live virus were added to the culture medium. After 48 h, supernatants of DCs cultures were harvested. Murine IL‒6 in harvested supernatants was measured using ELISA kits according to the manufacturer’s instruction. Supplied standards were used to generate standard curves.

### *In vivo* antitumor effect

CT-26 cells were enzymatically detached from culture flasks and counted. 5 × 10^6^ CT26 cells were resuspended in 100 μl PBS and injected into the subcutaneous space on the back of BALB/c mice (day 0). When tumors reached 5 mm in diameter, the mice were randomly divided into two groups (n = 10 per group). One group served as control and received one intratumoral administration of 100 μl saline. The other group was intratumorally administered with UV-Tianjin, once at a dose of 5 × 10^8^ in 100 μl saline on days 4, 8 and 12. Tumor size was measured in a blinded manner with slide calipers every other day, and survival of the animals was monitored. Tumor volume was calculated by using the following formula: tumor volume (mm^3^) = length × (width)^2^/2.

### Quantitative real-time RT-PCR

Tumors were removed at 24, 48, and 120 h after three injections of UV-Tianjin or saline. Total RNA was extracted using an RNeasy Mini Kit (Qiagen, Shenzhen, China). A total of 1 μg of RNA was reverse transcribed into cDNA with cDNA Reverse Transcription Kit (TaKaRa Bio, Dalian, China) and amplified by IQ SYBR Green Supermix (BioRad, Beijing, China) using a BioRad IQ5 icycler detection system under the following conditions: initial denaturation at 94°C for 3 min followed by 40 cycles of denaturation at 94°C for 30 s, annealing at 55°C for 30 s, and elongation at 72°C for 1 min. All procedures were carried out according to the manufacturer’s instructions. The 2^-ΔΔ*C*T^ equation was used to analyze the data. The data were presented as the fold change in gene expression normalized to an endogenous reference gene GAPDH (glyceraldehyde-3- phosphate dehydrogenase) and relative to the untreated control. ΔΔ*C*T = (*C*T, Target–*C*T, GAPDH) treated sample–(*C*T, Target–*C*T, GAPDH) untreated control. Primer pairs specific for murine CD4, CD8, CD11c, and GAPDH were synthesized by TaKaRa Bio (Dalian, China) and their sequences are listed in Table 1.

**Table 1:** Sequences of primers used in the real-time PCR amplifications

**Gene**	**Primer sequence**	**PCR product**
CD4	F:5’-TTGTGCATGTCACACATGAA-3’	550 bp
	R:5’-CCTGTGTTCCATGTAGTGGC-3’	
CD8	F:5’-GTCCGTTTCGCAAGGATGCT-3’	543 bp
	R:5’-CCTTCCTGTCTGACTAGCGG-3’	
CD11c	F:5’-GAAAGTGTGAAGTTGCTTCT-3’	500 bp
	R:5’-ACATGTCTGCTGCTACAGCT-3’	
GAPDH	F:5’-AGGCCGGTGCTGAGTATGTC-3’	530 bp
	R:5’-TGCCTGCTTCACCACCTTCT-3’	

### Immunohistochemical analysis of tumor tissue

Tumor tissues were harvested at 48 h after UV-Tianjin or saline injections for three successive days. All tumor tissues were fixed in 10% neutral buffered formalin and embedded in paraffin. Thin sections were stained with specific antibodies in combination with secondary antibodies according to the manufacturer’s instruction. The presence of brown granules on the surface of cells was defined as positive cell. The number of positive cells per section was counted in 10 random high-power microscopic fields (×400), and the percentage of positive cells (positive cells/total cells × 100%) was calculated [19]. Three discontinuous sections were selected from every specimen and those indexes were averaged. The final results were expressed as percentage mean ± standard deviation (SD).

### In situ detection of apoptotic cells in tumor tissue

At 48 h after three successive injections of UV-Tianjin or saline, tumor tissues were taken out from the mice. All tumor tissues were fixed in 10% neutral buffered formalin and embedded in paraffin. Analysis of apoptotic cells was performed by TUNEL apoptosis assay kit following the manufacturer’s direction. Images of the sections were taken by a fluorescence microscope (Olympus, Japan). Apoptotic index was calculated by dividing the number of TUNEL-positive cells by the total number of cells in the field.

### Statistical analysis

All data were expressed as means ± SD and analyzed by one way ANOVA test with Fisher’s adjustment, except for animal survival. Survival was analyzed with the Kaplan-Meier method and the log-rank test. A probability value of *P* < 0.05 was considered statistically significant.

## Results

### UV-Tianjin inhibited the growth of CT26 tumors and prolonged the survival of the treated mice

**Figure 1:**
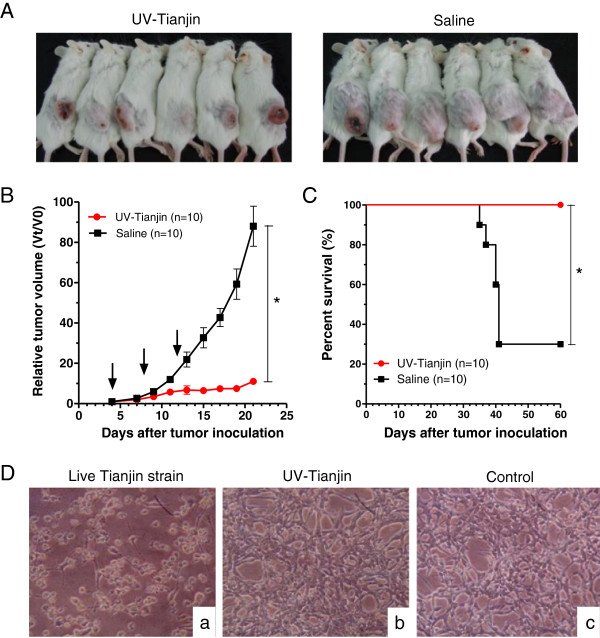
**Tumor growth inhibition by intratumor injection of UV-Tianjin. A** to **C**, 5 × 10^6^ CT26 cells were inoculated into subcutaneous space on the back of BALB/c mice. Particles of UV-Tianjin (5 × 10^8^ each) or saline were injected into tumors on days 4, 8, and 12. **A**, UV-Tianjin-treated or saline-treated BALB/c mice 3 weeks after tumor inoculation. The tumors treated by UV-Tianjin were much smaller than those treated by saline. **B**, The growth of UV-Tianjin-treated tumors was strongly suppressed compared with saline-treated tumors. Each point in the curve was expressed as the mean ± SD of the relative tumor volume Vt/V0 (where Vt denotes the tumor volume at test time point and V0 denotes the corresponding initial tumor volume at the beginning of treatment). ^*^P < 0.01 vs. control group. Arrows indicate the timing of injection. **C**, Survival curve of the mice bearing CT26 colon carcinoma following three consecutive injections of UV-Tianjin or saline, revealed a significant increasing of survival rate from 30% to 100% at day 60 by UV-Tianjin. ^*^P < 0.01 vs. control group. **D**, Microscopic view of CT26 cells infected with live Tianjin strain or UV-Tianjin on 4 dpi (days post infection). a, Live Tianjin strain infection produced an obvious cytopathic effect (CPE) characterized by cell rounding and detachment from the monolayer. b, UV-Tianjin infection can’t produce visible CPE. c, Healthy control. Photographs are shown at 100× magnification. Results are representative of three independent experiments.

To determine the antitumor effect of UV-inactivated Tianjin strain, virus particles of 5 × 10^8^ UV-Tianjin in a total volume of 100 μl were injected into CT26 tumors growing on the backs of syngeneic BALB/c mice. As shown in Figure 1A, B, and C, three consecutive injections of UV-Tianjin could obviously inhibit the growth of CT26 tumors and significantly prolonged the survival of the treated mice as compared to control group (*P* < 0.01). We also confirmed that cultured CT26 cells were refractory to direct contact with UV-Tianjin *in vitro* (Figure 1D).

### UV-Tianjin induced maturation and IL-6 release from murine dendritic cells

**Figure 2:**
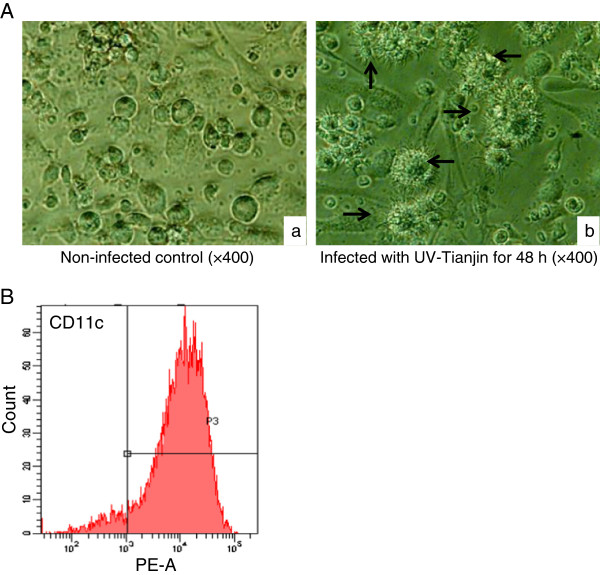
**Culture and identification of dendritic cells. A**, Morphological observation of dendritic cells cultured *in vitro* for 8 days. a, Non-infected DCs (×400). b, Infected with UV-Tianjin (MOI = 200) for 48 h (×400). Black arrows indicate mature DCs with many dendritic protrusions. **B**, Flow cytometric analysis of the expressing of CD11c molecule specific for DCs cultured *in vitro* for 6 days. Results are representative of three independent experiments.

**Figure 3:**
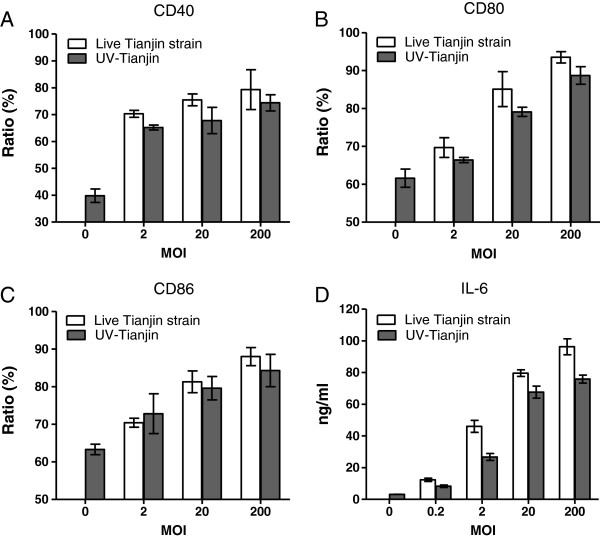
**Activation of murine dendritic cells by UV-Tianjin. A** to **C**, The expressions of CD40, CD80 and CD86 were measured by flow cytometry after 48 h of coculture with UV-Tianjin or live virus. Murine dendritic cells were dose-dependently maturated by virus particles to express CD40, CD80 and CD86, showing that UV-Tianjin retained the immunogenicity of live Tianjin strain. **D**, IL-6 secretion was measured in dendritic cell supernatants by ELISA at 48 h after coculture with UV-Tianjin or live virus. Amounts of IL-6 were slightly diminished after UV-Tianjin stimulation compared with corresponding dose of live virus. Columns represent mean value of triplicate samples and bars represent SD. Representative of three independent experiments.

On day 6 of culture, the suspended and loosely adherent cells derived from murine bone marrow were collected. Flow cytometric analysis to determine the purity of dendritic cells showed that more than 90% of the cells were positive for CD11c (Figure 2B). Then different doses of UV-Tianjin or live virus were incubated with these cells for 48 h and the maturation of DCs was observed under microscope. More mature DCs were seen in UV-Tianjin as well as live Tianjin strain group compared with control group, especially in high dose groups (Figure 2A). In addition, the expressing of markers of DCs maturation, CD40, CD80 and CD86 molecules were analyzed by flow cytometry. They were all dose-dependently increased by UV-Tianjin or live virus (Figure 3A, 3B and 3C). These results suggested that UV-Tianjin remained the antigenicity of live virus. Next, the amount of representative proinflammatory cytokine IL-6 released into DCs culture medium was measured by ELISA at 48 h after UV-Tianjin or live Tianjin strain treatment. The result showed that the secretion amount of IL-6 by UV-Tianjin was a little lower than that released in the presence of corresponding dose of live virus (Figure 3D).

### UV-Tianjin promoted dendritic cell and T-cell recruitment to tumor beds

**Figure 4:**
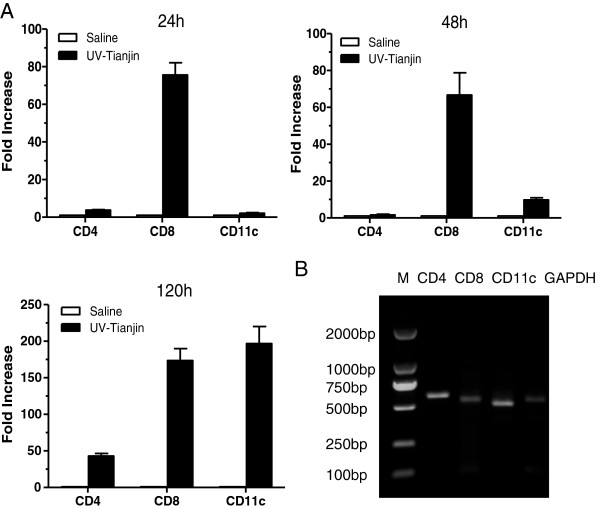
**Dendritic cell and T-cell infiltration into tumor *in vivo*. A**, mRNA expression quantified by real-time PCR in tumors at 24, 48, and 120 h after three successive injections of 5 × 10^8^ UV-Tianjin particles or saline. The expression levels of CD4, CD11c, especially CD8 in UV-Tianjin-treated tumors were significantly higher than those in corresponding saline-treated tumors at almost all time points. The data were normalized by GAPDH. Each value was calculated as fold increase relative to value of saline-treated group at the same time point. Columns, mean of triplicate samples; bars, SD. **B**, Electrophoretogram of RT-PCR amplification of CD4, CD8 and CD11c. Lane 1, 2000 bp DNA ladder; lane 2, CD4 (550 bp); lane 3, CD8 (543 bp); lane 4, CD11c (500 bp); lane 5, GAPDH (530 bp). One representative gel is shown. The data are representative of three independent experiments.

**Figure 5:**
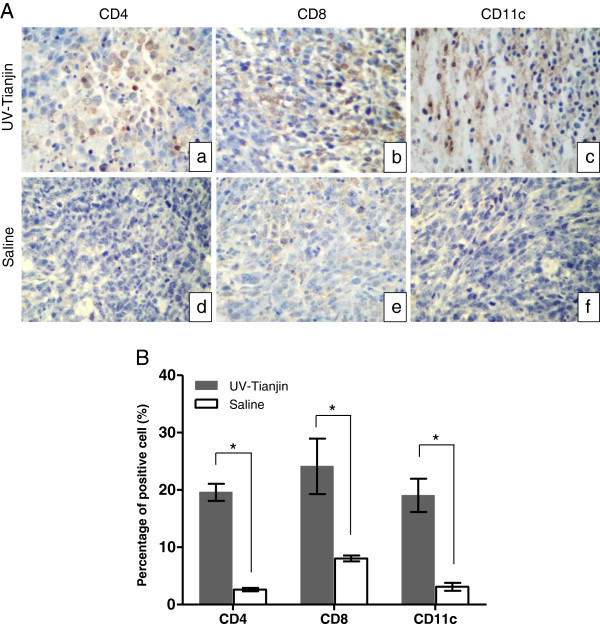
**Histologic findings of tumors. A**, Representative sections of tumors from UV-Tianjin-treated mice (a-c) or saline controls (d-f) (original magnification, ×400). CD4^+^ (a and d), CD8^+^ (b and e) or CD11c^+^ (c and f) cells were remarkably infiltrated into tumor tissues by UV-Tianjin. Tumor tissues were isolated at 48 h after UV-Tianjin or saline injection for three successive days. **B**, The percentage of CD4^+^, CD8^+^ and CD11c^+^ cells were shown as histogram bars. UV-Tianjin caused a significantly higher percentage of positive cells. ^*^P < 0.01, vs. control. The data are representative of three independent experiments.

To determine how host adaptive immune system reacts to UV-Tianjin *in vivo*, the mRNA expressions of CD11c as marker for DCs and of CD4 and CD8 for effector T cells were measured in tumors at 24, 48, and 120 h after UV-Tianjin injection in mice. The expression levels of CD11c, CD4, especially CD8, were significantly increased by UV-Tianjin at almost all time points compared with corresponding control group (Figure 4). And they remained on an upward trend until 120 h after treatment, which was a little different from the previous study that reported the expression levels of CD4 and CD11c peaked at 48 h in HVJ-E-treated tumors. In addition, immunohistochemical staining was done to evaluate DCs and T-cells infiltration into tumor tissues after UV-Tianjin treatment. The results showed that the percentage of CD4^+^, CD8^+^ and CD11c^+^ cells in UV-Tianjin-treated tumors were (19.60±1.49)%, (24.12±4.84)% and (19.05±2.91)%, respectively, which were much higher than those of corresponding control group (Figure 5A, 5B) (*P* < 0.01). It revealed that CD11c^+^, CD4^+^, and CD8^+^ cells were remarkably infiltrated into tumor by UV-Tianjin.

### UV-Tianjin induced the apoptosis of CT26 tumors in mice

**Figure 6:**
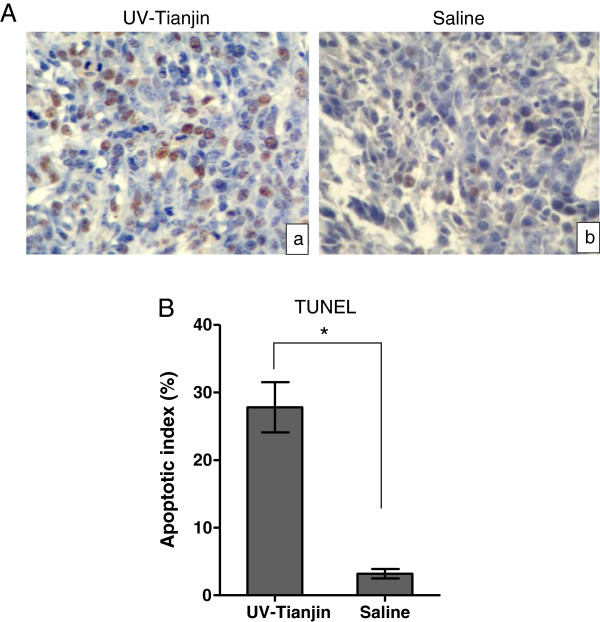
**The induction of apoptosis *in vivo *was evaluated by TUNEL staining.** Tumor tissues were harvested at 48 h after three injections of UV-Tianjin or saline. **A**, Representative photographs of the tumor sections (original magnification, ×400). Brown staining indicates TUNEL-positive nuclei and blue staining counterstained with hematoxylin indicates TUNEL-negative nuclei. a, UV-Tianjin-treated group; b, Saline-treated group. **B**, Percentage of TUNEL positive nuclei was calculated. Each bar represents the “apoptotic index”, expressed as mean ± SD. UV-Tianjin caused a significantly higher percentage of TUNEL-positive apoptotic cells. ^*^P < 0.01 vs. control. The data are representative of three independent experiments.

To confirm the ability of UV-Tianjin to elicit apoptosis *in vivo*, in situ TUNEL staining was carried out on tissue sections of tumors excised at 48 h after UV-Tianjin injection. As shown in Figure 6A, UV-Tianjin-treated tumor showed significantly more apoptotic cells (with brown nuclei) compared with control group. The apoptosis index of UV-Tianjin-treated group [(27.8 ± 3.7)%] was much higher than that of saline-treated group [(3.2 ± 0.7)%] (Figure 6B, *P* < 0.01). These results suggested that the increased tumor cell apoptosis was also responsible for the antitumor effect of UV-Tianjin *in vivo*.

## Discussion

Viral antitumor effect involves in many kinds of modes including directly killing the tumor cells, inducing tumor cells apoptosis and stimulating the immune response of host. Previous studies have shown that UV-inactivated, replication-defective Sendai virus particles (HVJ-E) could induce antitumor immunity through both the activation of CTL and natural killer (NK) cells and the suppression of regulatory T cells [11,12]. Furthermore, HVJ-E could also induce cancer-selective apoptosis via the upregulation of TRAIL and Noxa downstream of the RIG-I/MAVS pathway [20].

Sendai virus strain Tianjin is a novel genotype of Sendai virus. In this study, we applied UV-inactivated, replication-defective Tianjin strain (UV-Tianjin) on a mouse model of CT26 colon carcinoma. As expected, UV-Tianjin markedly inhibited the growth of CT26 tumors and significantly prolonged the survival of the treated mice. UV-Tianjin is unable to replicate in cells because the genome is destroyed by UV irradiation. Moreover, our results showed that UV-Tianjin was non-toxic to murine CT26 cancer cell lines (Figure 1D). Therefore, the mechanism of antitumor activity for UV-Tianjin was probably not associated with direct UV-Tianjin cytotoxicity.

DCs are potent antigen presenting cells (APCs) that play a crucial role in regulating the adaptive immune response. DCs process and present antigens to activate both CD4^+^ and CD8^+^ T cells. Thus activation of tumor antigen-specific CD8^+^ CTL and CD4^+^ T helper cells generates antitumor immune response. In addition, mature DCs can express high levels of costimulatory molecules CD40, CD80 as well as CD86 and secret IL-6 and other Thl type cytokines which induce Thl cell immunity [21]. Recently, the application of DCs in immunotherapy of tumor has become a research focus in the field of initiative specific immunotherapy of tumor [22,23]. In the present study, we examined whether UV-Tianjin could activate DCs and induce tumor-specific immunity. We found that murine DCs underwent dose-dependent maturation by UV-Tianjin and the amount of IL-6 released from DCs was slightly diminished after UV-Tianjin stimulation as compared to live virus *in vitro*. Furthermore, DCs and effector T-cell recruitment to tumors after UV-Tianjin treatment was suggested from the significant increase of CD11c, CD4, and CD8 mRNA expression. Remarkable infiltrations of DCs and CD4^+^ and CD8^+^ T cells into UV-Tianjin-treated tumors were also confirmed in immunohistochemical studies. At present, the precise molecular and cellular mechanisms of activation of DCs by SeV are not well-understood. HVJ-E is known to induce maturation of dendritic cells in a Toll-like receptor-independent manner [24]. In addition, a recent report indicated that HVJ-E could induce cytotoxic T cells against cancers and inhibit regulatory T cells by IL-6 expression in dendritic cells [11]. These facts support the induction of anticancer immunity in colon carcinoma by UV-Tianjin treatment.

Apoptosis is ubiquitous in the majority of tumor cells, and is important in the genesis and progression of tumors. Previous studies have demonstrated that some viruses typically inhibit tumors by inducing apoptosis of sensitive tumor cells [20,25,26]. In our study, we also performed TUNEL assay to observe whether cell apoptosis was responsible for the antitumor effect of UV-Tianjin *in vivo*. Results showed that the apoptotic index of treatment group was significantly higher than that of control group. As far as we know, the molecular mechanism of HVJ-E–induced apoptosis in cancer cells has not been fully investigated. Recent data suggested that TNF-related apoptosis-inducing ligand (TRAIL) and Noxa represented promising target molecules for cancer cell-selective apoptosis when the retinoic acid–inducible gene I/mitochondrial antiviral signaling protein (RIG-I/MAVS) signaling pathway was activated [20]. In view of the lack of replicative ability and non-toxicity to cultured CT26 cells of UV-Tianjin, we hypothesized that the possible mechanism of UV-Tianjin–induced apoptosis might be that UV-Tianjin RNA fragments are recognized by RIG-I and then the activation of the RIG-I/MAVS pathway triggers cancer cell-selective apoptosis. Further study is required to provide more proof.

## Conclusions

Taking all these results together we demonstrated that UV-Tianjin alone had antitumor activity against colon carcinoma. Mechanistically, not only antitumor immune response through DCs and T cells but also tumor cell apoptosis was responsible for this antitumor effect. But the detailed mechanism requires to be cleared. To our knowledge, for the first time we reported the growth inhibitory effect of UV-Tianjin on colon carcinoma by inducing antitumor immune response and cell apoptosis. UV-Tianjin may offer important contributions for the development of a novel drug to prevent and cure human cancers including colon carcinoma in the future.

## Competing interests

The authors declare that they have no competing interests.

## Authors’ contributions

LS conceived the study, analyzed and interpreted data, drafted the manuscript. JC and QZ carried out ELISA assay and flow cytometric analysis. ML, PG and JH conducted TUNEL assay and immunohistochemistry staining. ZH and MS performed real time RT-PCR. HT conceived the study, performed statistical analysis and helped to draft the manuscript. All authors read and approved the final manuscript.
